# High-Efficient Production of (*S*)-1-[3,5-Bis(trifluoromethyl)phenyl]ethanol via Whole-Cell Catalyst in Deep-Eutectic Solvent-Containing Micro-Aerobic Medium System

**DOI:** 10.3390/molecules25081855

**Published:** 2020-04-17

**Authors:** Zhiren Zhu, Shunde Bi, Ning Ye, Pu Wang

**Affiliations:** College of Pharmaceutical Science, Zhejiang University of Technology, Hangzhou 310014, China; zhuzhiren619@163.com (Z.Z.); bishundezjut@163.com (S.B.); itsukiyening@gmail.com (N.Y.)

**Keywords:** cofactor regeneration, natural deep-eutectic solvents (NADESs), oxygen-deficient environment, ratio of substrate/catalyst (S/C)

## Abstract

The ratio of substrate to catalyst (S/C) is a prime target for the application of asymmetric production of enantiomerically enriched intermediates by whole-cell biocatalyst. In the present study, an attractive increase in S/C was achieved in a natural deep-eutectic solvent (NADES) containing reaction system under microaerobic condition for high production of (*S*)-1-[3,5-bis(trifluoromethyl)phenyl]ethanol ((*S*)-3,5-BTPE) with *Candida tropicalis* 104. In PBS buffer (0.2 M, pH 8.0) at 200 rpm and 30 °C, 79.5 g (Dry Cell Weight, DCW)/L *C. tropicalis* 104 maintained the same yield of 73.7% for the bioreduction of 3,5-bis(trifluoromethyl)acetophenone (BTAP) under an oxygen-deficient environment compared with oxygen-sufficient conditions, while substrate load increased 4.0-fold (from 50 mM to 200 mM). Furthermore, when choline chloride:trehalose (ChCl:T, 1:1 molar ratio) was introduced into the reaction system for its versatility of increasing cell membrane permeability and declining BTAP cytotoxicity to biocatalyst, the yields were further increased to 86.2% under 200 mM BTAP, or 72.9% at 300 mM BTAP. After the optimization of various reaction parameters involved in the bioreduction, and the amount of biocatalyst and maltose co-substrate remained 79.5 g (DCW)/L and 50 g/L, the S/C for the reduction elevated 6.3 times (3.8 mM/g versus 0.6 mM/g). By altering the respiratory pattern of the whole-cell biocatalyst and exploiting the ChCl:T-containing reaction system, the developed strategy exhibits an attractive potential for enhancing catalytic efficiency of whole-cell-mediated reduction, and provides valuable insight for the development of whole-cell catalysis.

## 1. Introduction

**Figure 1:**
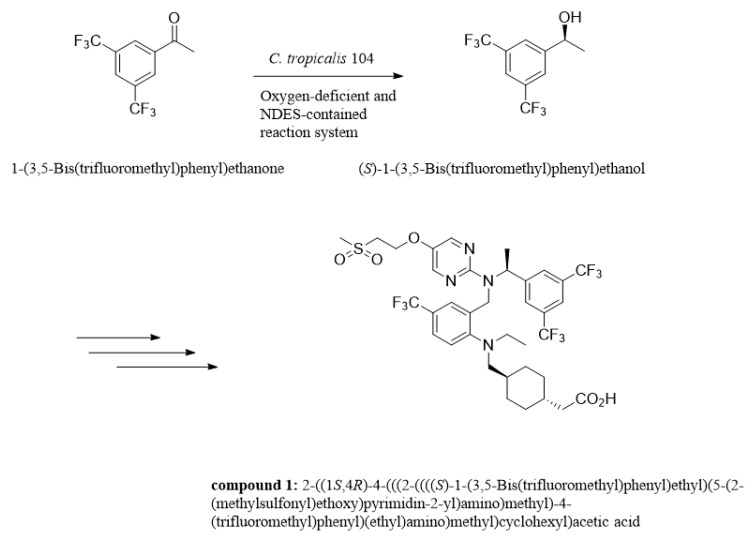
Sketch of synthesis compound **1** of lipid-lowering agent.

Asymmetric reduction is a direct and vital approach to acquiring enantiomerically enriched alcohols in chemical catalysis or biocatalysis, which has received much attention and been explored for decades [1,2,3]. Optically active isomer of 3,5-bis(trifluoromethyl)phenyl ethanol (3,5-BTPE) has been exhibited to be versatile in pharmaceutical synthesis, such as (*R*)-[3,5-bis(trifluoromethyl)phenyl]ethanol ((*R*)-3,5-BTPE) for compounding the NK1 antagonists [4,5], and (*S*)-3,5-BTPE is a crucial intermediate of lipid-lowering agent (compound **1**, shown in Figure 1), which is capable of reducing the amount of PCSK9 protein and increasing the amount of low density lipoprotein-receptor [6,7]. Two primary methods of acquiring (*S*)-3,5-BTPE are known: chemical catalysis and biocatalysis. Compared to chemical synthesis, a biological approach exploits a cell or enzyme instead of a rare-metal-contained compound as catalyst, and avoids involving a good deal of organic/inorganic hydrogen donors, or even insecure H_2_ gas for hydrogen donors [8,9,10,11]. Obviously, acquiring chiral alcohols via biocatalysis conforms to the “green chemistry” principle. In consideration of the convenience and cost-effectiveness for practical application, the whole-cell catalyst holds immense potential compared to an enzyme catalyst [10,11,12].

Furthermore, in the whole-cell catalysis for bioprocess, the whole cell could be in growing and proliferation status [13,14], such as the biocatalysis of 2,3-butanediol production by *B. licheniformis* DSM 8785, or in resting status [12,15], such as (*S*)-3,5-BTPE production by *Rhodococcus erythropolis* XS1012. The cells will be in a buffer medium to be non-growing status for less metabolism and the enzyme with the ability for catalysis remains in the cell [16]. Thus, for the whole-cell catalysis in bioprocess, the resting status cell as catalyst will probably facilitate the bioprocess and promote catalytic efficiency.

For the potential medicinal value of (*S*)-3,5-BTPE, the biocatalytic asymmetric reduction of BTAP to (*S*)-isomer has been examined by a few groups [11,15], and the research on bioprocesses via whole-cell catalysts are few [12,15]. In the present study, *C. tropicalis* 104 was employed as a whole-cell catalyst in (*S*)-3,5-BTPE production, and this bioreduction was used as a model reaction. The isolate of *C. tropicalis* 104 was obtained in our previous work with excellent enantioselectivity [12].

For more efficient production by biocatalytic process, there are many process metrics to control, such as substrate conversion efficiency, catalyst recycling or ratio of substrate to catalyst (S/C) [17,18,19]. For example, a high S/C ratio is one of significant issues in highly efficient preparation via biocatalysis. Generally, an unsatisfactory S/C ratio is a bottleneck for scaled-up production in whole-cell catalysis compared with chemical catalysis and enzymatic catalysis [8,10,11,15]. There are several measures associated with the S/C ratio enhancement, such as improvement of biocatalyst activity, promoting cofactor regeneration and enhancement of membrane permeability in cells, or reducing inhibition of the substrate [20,21,22,23]. Many ways with respect to optimization of key parameters related to the S/C ratio have been exhibited [24,25,26,27]. For instance, by enhancing CO_2_ content to improve the photosynthesis of recombinant *Synechocystis elongatus* PCC 7942, the capacity of cofactor regeneration in the biocatalyst was efficaciously enhanced [25]. Altering the composition of air has obvious effects on photoautotrophic microorganisms in biocatalysis, but research on asymmetric reduction of aromatic ketone by nonphotoautotrophic microorganisms has been infrequent. In order to decline substrate inhibition to biocatalyst, generally, a water-organic biphase reaction system was established for substrate transient storage and separating the cells from substrate [28]. Recently, using ionic liquids (ILs) or deep-eutectic solvents (DESs) is a hot spot of research for enhancing mass-transfer efficiency. An IL 1-ethyl-3-methylimidazolium tosylate (Emim:TOS) containing reaction medium was established with the conversion yield doubled, because the IL in the reaction medium increased the mass-transfer efficiency for reducing aggregation of the *R. erythropolis* MA7213 in bioprocess [29]. Zhang, et al. reported that, by the treatment of *Escherichia coli* cells with choline chloride:urea to improve membrane permeability has led to the boost of the binding between intracellular enzyme and substrate [23]. Up to now, the function of deep-eutectic solvent (DES) or NADES for whole-cell catalysis was prevailingly focused on the enhancement of cell membrane permeability or substrate solubility in aqueous medium [21,23,30,31].

In this study, we examined the impact of oxygen supply on the model reaction for (*S*)-3,5-BTPE production mediated by *C. tropicalis* 104 resting cells, and found that performing the bioreduction under an oxygen-deficient environment led to an appealing improvement in the ratio of S/C. Moreover, by the introduction of ChCl:T into the reaction system, the bioreductive efficiency was evidently improved. It is the first report on establishing and exploiting an efficient integrated strategy of changing the respiratory pattern of a whole-cell catalyst, and constructing a multifunctional NADES-containing reaction system for challenging the asymmetric synthesis of chiral alcohols under high substrate loading. The developed mixed-strategy also provides a valuable insight for the scaled-up development of asymmetric reduction by a whole-cell biocatalyst.

## 2. Results and Discussion

### 2.1. Oxygen Control Means Selecting and Effect on (S)-3,5-BTPE Production by C. tropicalis 104

#### 2.1.1. Developing an Oxygen Control Proposal for (*S*)-3,5-BTPE Production by *C. tropicalis* 104

**Figure 2:**
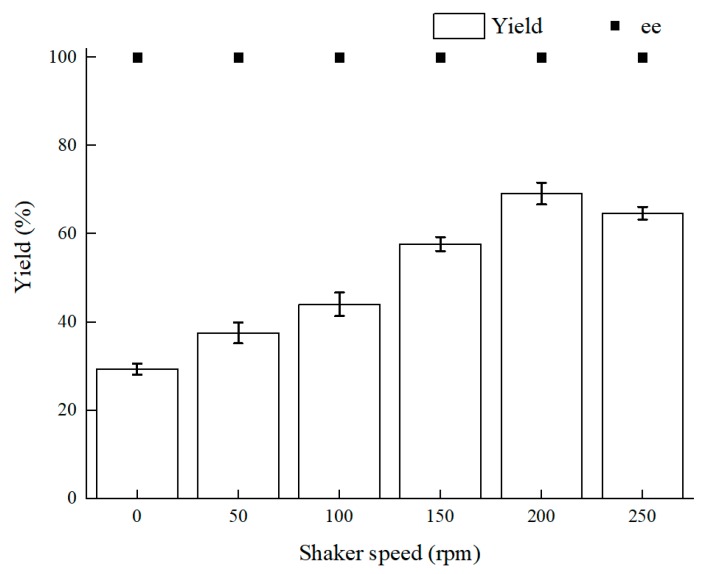
Effect of different shaker speeds on yield and product ee value. Reaction conditions: 10 mL potassium phosphate buffer (0.2 M, pH 8.0), 300 g/L cells (wet weight), 50 mM 3,5-bis(trifluoromethyl)acetophenone (BTAP), 50 g/L maltose, 30 °C, reaction for 30 h.

Oxygen content makes a great difference to intracellular redox homeostasis such as NADH (Nicotinamide adenine dinucleotide) content. Using a microaerophilic environment to keep intracellular redox homeostasis has a signal effect on biocatalysis [14,24]. The shaker speed is relevant to oxygen supply and mass-transfer efficiency. According to the results in Figure 2, 200 rpm was the optimal shaker speed. First, as the rotating speed lowered, the yield reduced and the more biocatalyst precipitates could be observed. Because the mass-transfer efficiency is dependent on shaker speed, diffusion of the substrate at a lower rotating speed reduced the mass-transfer efficiency. Therewith, mass-transfer efficiency led to less contact of the biocatalyst and BTAP. Second, as the shaker speed rose over 200 rpm, the yield of (*S*)-3,5-BTPE did not keep increasing and even declined slightly, and the reasons for this result could be as follows: (1) there was an immoderately high physical force from high speed of the shaker, and cells could not endure the force and the cell integrity might be damaged [32]; (2) as the rotating speed rose over 200 rpm, superfluous oxygen within cells might give rise to oxidative stress, such as damage of the enzyme or DNA [33].

**Figure 3:**
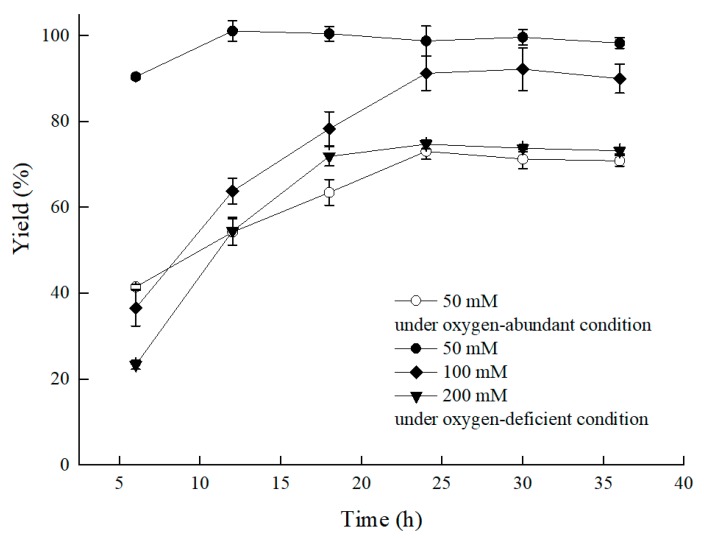
Effect of substrate concentration on the asymmetric reduction of BTAP under oxygen-deficient environment. Reaction conditions: 10 mL potassium phosphate buffer (0.2 M, pH 8.0), 300 g/L cells (wet weight), a certain amount of BTAP, 50 g/L maltose, 30 °C, 200 rpm, reaction for 36 h. Except control group, the others were all under oxygen-deficient environment.

Nevertheless, we were inspired by the bioprocess that used the *Bacillus* sp.-mediated efficient reduction of acetoin to 2,3-butanediol by means of reducing the agitation speed or adding vitamin C/E [13,14]. To raise catalytic efficiency, the *C. tropicalis* 104-mediated asymmetric reduction of BTAP proceeded by limiting oxygen supply by settling the bioprocess in a conical flask with a stopper, and it worked. As the results showed in Figure 3, compared to the group without the stopper, a higher ratio of S/C was achieved under the oxygen-deficient condition, and the bioreductive period was shortened. The yield of the group with the stopper reached 90.5% at 0.6 mM/g of S/C in 6 h under 50 mM BTAP and 300 g/L cell concentration (79.5 g/L DCW). The product enantiomer excess (ee) for (*S*)-3,5-BTPE was over 99.9%. Comparatively, a yield of only 41.5% was observed under the oxygen-sufficient condition, with a product ee value over 99.9%. Furthermore, benefiting from the oxygen-deficient condition, a 73.7% yield was obtained at 200 mM BTAP, and a 4.0-fold increase in S/C for BATP reduction by *C. tropicalis* 104 in the energy-saving mode was evident.

However, although the efficiency of (*S*)-3,5-BTPE production via *C. tropicalis* 104 was enhanced by limiting the oxygen supply, the mechanism of the effects of above operations on the cells in the (*S*)-3,5-BTPE production was unknown.

#### 2.1.2. Effect of Limited Oxygen on *C. tropicalis* 104 in Bioprocess

In an attempt to explain the remarkable effect of microaerobic conditions on improving the catalytic efficiency of *C. tropicalis* 104 mediated (*S*)-3,5-BTPE production, the intracellular NAD(P)H concentration of *C. tropicalis* 104 was detected because of the relevance between cofactor cycle and asymmetric reduction of prochiral ketone to chiral alcohol [34,35]. Firstly, the cofactor specificity of *C. tropicalis* 104 cells for BTAP reduction was estimated by the assay of OD_340 nm_ variation as the indicator of NAD(P)H content in a cell-free extract from *C. tropicalis* 104 [36], and the results were illustrated in Table 1. The carbonyl reductase derived from *C. tropicalis* 104 cells for BTAP reduction emerged cofactor preference to NADH. As the results of intracellular NADH concentration shown in Table 2, compared with the group under the oxygen-sufficient condition, it was detected that the NADH concentration increased by 2.2 times under the oxygen-deficient condition.

First of all, NADH, which is consumed primarily in respiration to generate ATP, was mainly produced by the catabolism in cytoplasm and the tricarboxylic acid (TCA) cycle [37,38]. Secondly, as research has shown [39], when in an oxygen-abundant environment, the water-forming NADH oxidase (noxE) within the exogenous gene of noxE expressible recombinant *Candida tropicalis* No. 121 increased and would consume NADH. Then, for the explanation of Table 2, there might be a conjecture: the crude *C. tropicalis* 104 in our study might possess and be able to express the gene of noxE or an analogue naturally, and noxE or an analogue within the cell might be shut down for there was little oxygen for accepting hydride ions, as crude *C. tropicalis* 104 was settled in an oxygen-abundant environment. Spontaneously, as cofactor NADH content increased, the BTAP reduction to (*S*)-3,5-BTPE was further facilitated by carbonyl reductase in crude *C. tropicalis* 104. The other factors also may be related to NADH concentration enhancement. According to the research [40], while the oxygen content was reduced in cell, less NADH from glycolysis would be transferred into the electron transport chain in mitochondria, and the carbonyl reductase might have more available NADH for reduction.

In addition, the approach of exploiting crude *C. tropicalis* 104 under oxygen-deficient conditions to alter cell energy metabolism for enhancing catalytic efficiency was more convenient and cost-effective compared to the introduction of exogenous cofactor regeneration, direct addition of an exogenous cofactor or other cosubstrate to enhance cofactor regeneration [34,41].

Additionally, our results showed that altering the whole-cell biocatalyst’s intracellular redox homeostasis is an effective way to improve biocatalyst productivity in other crude strain. As shown in Table 3, when the reaction was under an oxygen-deficient environment compared with an oxygen-abundant environment as control, the yield of catalysis via *C. tropicalis* 104 increased from 68.8% to 91.3% with over 99.9% ee, and the yield of catalysis via *G. candidum* ZJPH1704 rose from 2.5% to 60.4% with ee increasing from 66.9% to over 99%. However, the yield of catalysis via *R. erythropolis* XS1012 was slightly decreased. The oxygen-deficient environment had positive effects on *C. tropicalis* 104 and *Geotrichum candidum* ZJPH1704, but a lesser effect on *Rhodococcus erythropolis* XS1012. Therefore, it seems that noxE [39] or isozyme was present in both *C. tropicalis* 104 and *G. candidum* ZJPH1704, but *R. erythropolis* XS1012 had a correlation with altering the whole-cell biocatalyst’s intracellular redox homeostasis in BTAP reduction to (*S*)-3,5-BTPE.

### 2.2. NADESs Selection and Effect on (S)-3,5-BTPE Production by C. tropicalis 104

#### 2.2.1. Effect of Various NADESs on Membrane Permeability of *C. tropicalis* 104

**Figure 4:**
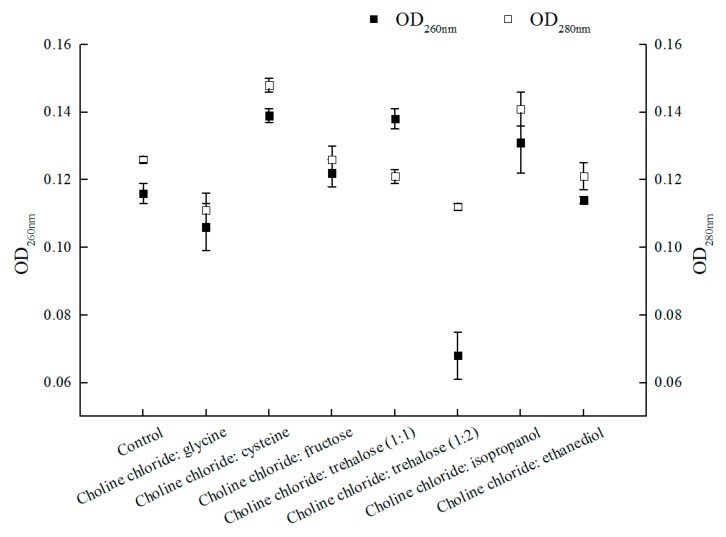
Effect of various NADESs on membrane permeability of *C. tropicalis* 104 cells. Reaction conditions: 0.2 M phosphate buffer (pH 8.0), 1% (*w*/*v*) various NADESs, and 300 g/L *C. tropicalis* 104, 30 °C and 200 rpm for 6 h.

Strengthening mass-transfer also exerted impact on catalytic efficiency. Some positive results were observed with the enhanced membrane permeability by introducing ionic liquids or DESs in the reaction medium for bioconversion catalyzed by microbial cells [30,31]. However, exploiting NADESs with multifunction in aqueous reaction system was rarely explored [30,42]. In this research, seven choline chloride-based NADESs were evaluated for their performances in the asymmetric reduction of BTAP. As shown in Figure 4, choline chloride:cysteine, ChCl:T (1:1, molar ratio), and choline chloride:isopropanol all increased the membrane permeability of *C. tropicalis* 104 in different degrees. Among these three NADESs, the values of OD_260 nm_ and OD_280 nm_ were different, especially ChCl:T (1:1)-added group for the increasing of OD_260 nm_ and declining OD_280 nm_, which were opposite to other groups. Refer to the OD_260 nm_ value in connection with nucleic acid content, it was found that the leak of nucleic acid was obvious in choline chloride:cysteine-added group, ChCl:T (1:1)-added group and choline chloride:isopropanol-added group, while the leaks of nucleic acid in other groups were slight or null. As the value of OD_280 nm_ was concerned with protein content, the leaks of protein in choline chloride:cysteine- and choline:isopropanol-added groups were apparent, while the leaks of protein in other groups were lower than the control. Nevertheless, when undue leak of nucleic acid or intracellular protein occurred, the cells would be viability-declined or even be killed [30]. It is crucial to balance the membrane permeability and viability of the cells for biocatalysis.

Interestingly, the other groups of the results did not affect or even decrease the membrane permeability, especially for choline chloride:glycine and ChCl:T (1:2). There were some matters relevant to that. For choline chloride:glycine, as a component of the NADES, glycine could protect cells and maintain membrane potential, although the definite mechanism still remains unclear [43,44]; As for ChCl:T (1:2 in molar ratio), while the trehalose proportion in this NADES went up from 1/2 to 2/3, both the values of OD_260 nm_ and OD_280 nm_ declined due to the predominant protection effect of trehalose. Based on the results showed above, the NADES ChCl:T (1:1) was capable of not only providing the enhancement of membrane permeability, but also preventing the leak of intracellular proteins. Moreover, trehalose can restore acid-denatured myoglobin to its native structure [45,46]. Trehalose in the introduced NADES ChCl:T (1:1) might provide protection to the protein (for example, an enzyme). Our results provide some valuable insight for the multifunction of ChCl:T (1:1) NADES.

#### 2.2.2. Effect of Various NADESs on Cell Viability of *C. tropicalis* 104

**Figure 5:**
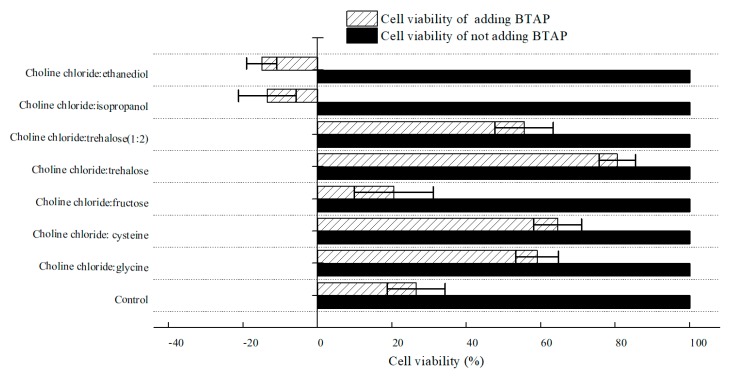
Effect of various NADESs on cell viability of *C. tropicalis* 104 cells. Conditions: 300 g/L cells (wet cell weight) were exposed to buffer systems comprising 1% (*w*/*v*) of various NADESs and potassium phosphate buffer (0.2 M, pH 8.0), with and without 200 mM BTAP. The cell viability of *C. tropicalis* 104 cells was taken as 100% in buffer systems without the addition of BTAP.

To estimate NADESs’ impact on the cell viability of *C. tropicalis* 104, cell viability was examined by means of detecting the residual glucose content after hatching with various NADES-treated *C. tropicalis* 104 cells. As shown in Figure 5, it seemed that all tested NADESs did not inhibit cell viability. With the addition of BTAP, however, cell viability declined in varying degrees. Obviously, the control group demonstrated that BTAP was highly toxic to the biocatalyst. Cell viability of groups treated by choline chloride:glycine, choline chloride:cysteine, ChCl:T (1:1), and ChCl:T (1:2) was higher than the control. Moreover, among all tested NADESs, ChCl:T (1:1) group gave the best cell viability, and approximately 3.0-fold higher than the control with 200 mM BTAP. However, though the choline chloride:cysteine increased cellular membrane permeability most among the DESs, the cell viability of its group was not the best. So, while the cellular membrane permeability was overdoing, the cell viability would decrease for damaging structures of cells. As seen in Figure 4, with the increased trehalose proportion in NADES, the protection effect of NADES will surpass its surfactant function. Because of that, ChCl:T (1:2) was blocked outside the cells for its poor capacity of increasing membrane permeability, and the enzymes in cells might be vulnerable to BTAP without the protection of trehalose. Thus, if the cellular membrane permeability was low, the cell viability surely would not be reduced by it, but the catalytic efficiency would decrease most likely for a reduced mass transfer rate of substrate. Hence, to be satisfactory to both sides of NADESs on membrane permeability and cell viability, the ChCl:T (1:1) was the most compatible among the examined NADESs.

#### 2.2.3. Effect of NADESs on (*S*)-3,5-BTPE Production with *C. tropicalis* 104 under Oxygen-Deficient Environment

**Figure 6:**
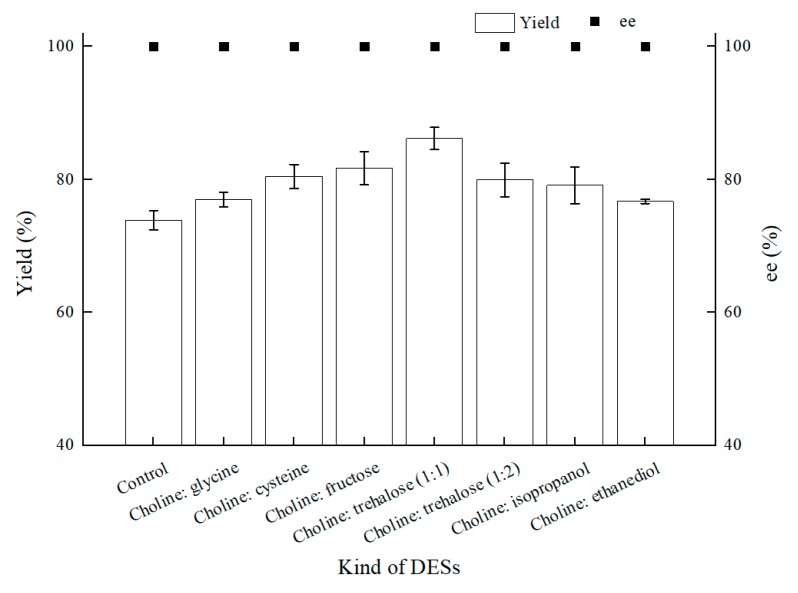
Effect of various NADESs on yield and product ee under oxygen-deficient environment. Reaction conditions: 10 mL potassium phosphate buffer (0.2 M, pH 8.0), 300 g/L cells (wet cell weight), 200 mM BTAP, 50 g/L maltose, 30 °C, 200 rpm, reaction for 24 h. The amount of NADESs was all added by 1% (*w*/*v*).

Encouraged by the foregoing positive effects of difunctional ChCl:T (1:1) for BTAP asymmetric reduction with *C. tropicalis* 104, ChCl:T (1:1) was determined for establishing a NADES-containing reaction system, and we combined this novel reaction medium with “energy-saving” mode in *C. tropicalis* 104 bioconversion. To verify the results of NADESs selection, as shown in Figure 6, seven NADESs were assessed for their performances in BTAP bioreduction. Except the choline chloride:ethanediol group, which gave roughly the same yield as control, the others increased the yields by different degrees owing to the enhanced membrane permeability or remaining cell viability, and the ChCl:T (1:1) group turned out to be the best.

**Figure 7:**
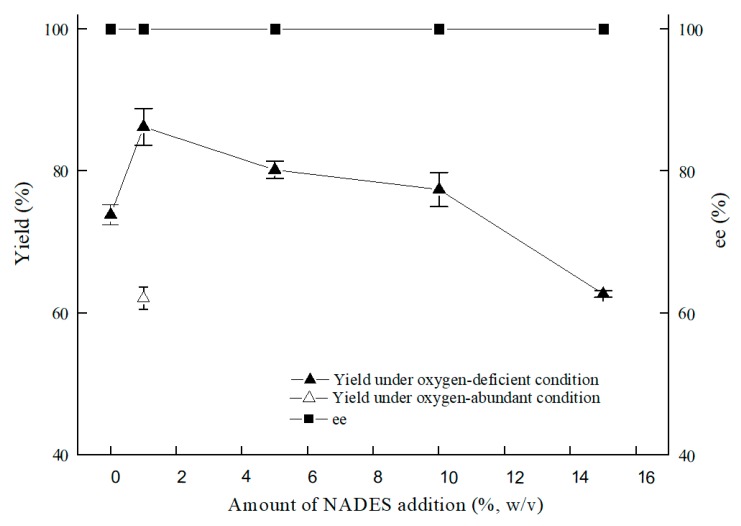
Effect of ChCl:T (1:1) amount on yield and product ee under oxygen-deficient or oxygen-abundant environment. Reaction conditions: 10 mL potassium phosphate buffer (0.2 M, pH 8.0), 300 g/L cells (wet cell weight), 200 mM BTAP, 50 g/L maltose, adding certain amount (*w*/*v*) of ChCl:T (1:1), 30 °C, 200 rpm, reaction for 24 h.

**Figure 8:**
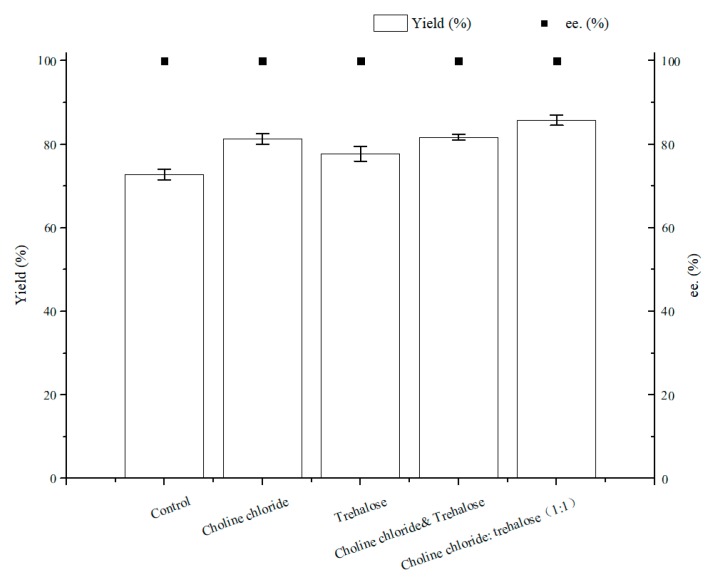
Effect of different components of ChCl:T (1:1) on yield and product ee under oxygen-deficient environment. Reaction conditions: 10 mL potassium phosphate buffer (0.2 M, pH 8.0), 300 g/L cells (wet cell weight), 200 mM BTAP, 50 g/L maltose, 30 °C, 200 rpm, reaction for 24 h. Except “control”, the other groups adding 1% (*w*/*v*) choline chloride, 1% (*w*/*v*) trehalose or 1% (*w*/*v*) ChCl:T (1:1), respectively.

As illustrated in Figure 7, the optimal content for NADES ChCl:T (1:1) addition was 1% (*w*/*v*). The reason for the result of the 1% (*w*/*v*) amount of ChCl:T (1:1) might be that while the dosage of ChCl:T (1:1) increased unduly, the membrane permeability of the cells probably went beyond the suitable level and might have weakened the vitality of the cells, or the protection from ChCl:T (1:1) overdid. As Figure 8 showed, the effect of ChCl:T (1:1) on (*S*)-3,5-BTPE production was better than the effects of one-component of ChCl:T (1:1) or a mixture of them. And it was probably because the presence of intermolecular forces [47] made the choline chloride and trehalose bind, and the trehalose would be able to enter the cell easily to protect the structure in the cell. What’s more, the data in Figure 7 expounded that when *C. tropicalis* 104 resting cells were situated monolithically in an oxygen-deficient condition, or catalyzed in a 1% (*w*/*v*) NADES-containing reaction medium, the yields of 73.8% or 62.1% were obviously lower than the yield of 86.2% under both of the conditions above.

**Figure 9:**
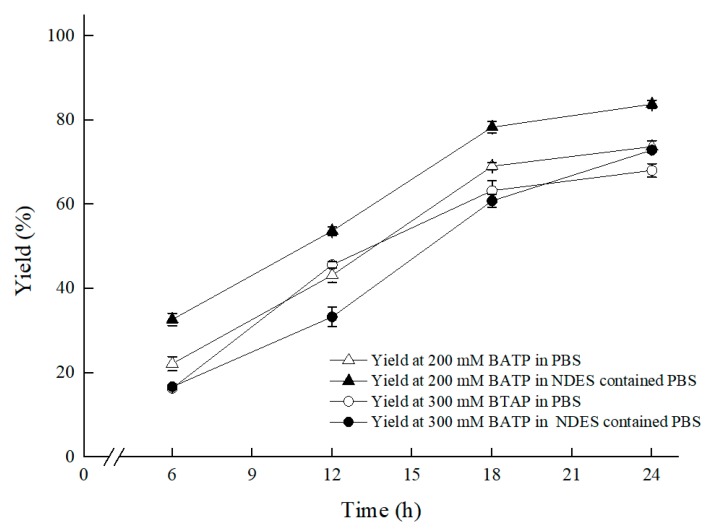
Effect of substrate concentration on the asymmetric reduction under oxygen-deficient environment with NADES-containing system. Reaction conditions: 10 mL potassium phosphate buffer (0.2 M, pH 8.0), 300 g/L cells (wet cell weight), a certain amount of BTAP, 50 g/L maltose, 30 °C, 200 rpm, reaction for 24 h. Except “200 mM BTAP” group and “300 mM BTAP” group, the “ChCl:T (1:1) containing and 200 mM BTAP” group and “ChCl:T (1:1) containing and 300 mM BTAP” group were under oxygen-deficient environment and reacted in NADES-containing system.

Furthermore, under the conditions of 30 °C and 200 rpm shaker speed, and in the established ChCl:T (1:1) containing reaction medium, which was composed of 300 g/L (wet cell weight) cell, 50 g/L maltose, 1% (*w*/*v*) ChCl:T (1:1), 0.2 M PBS (pH 8.0), as Figure 9 showed, BTAP concentration further upped from 200 mM to 300 mM, with a yield of 72.9%, which benefited from enhanced membrane permeability and declining cytotoxicity of BTAP to the biocatalyst based on the multifunctional ChCl:T (1:1). For the presence of ChCl:T (1:1) with multifunction of enhancing membrane permeability for mass transfer efficiency and protecting cells from 3,5-BTAP cytotoxicity, the ratio of S/C was further enhanced from 2.5 mM/g to 3.8 mM/g.

## 3. Materials and Methods

### 3.1. Materials

The source of BTAP (purity > 99%) was Beijing Golden Olive Co., Ltd., China. (*R*)-3,5-BTPE (purity > 99%) and (*S*)-3,5-BTPE (purity > 99%) were provided by Capot Chemical Co., Ltd., Hangzhou, China. NADH (BC grade), NADPH (BC grade), ADH (activity ≥300 U/mg protein) and MTT (BC grade) were purchased from Sangon Biotech Co., Ltd., Shanghai, China. Phenazine ethosulfate (PES, BR grade) was bought from Yuanye Shengwu Co., Ltd., Shanghai, China. DESs (purity > 98%) were synthesized and supplied by Shanghai Fujie Chemical Co., Ltd., China. All other reagents and chemicals were obtained from commercial sources.

### 3.2. Methods

#### 3.2.1. Microorganism and Cultivation

*C. tropicalis* 104 cells were incubated in seed medium for 12 h at 200 rpm and 30 °C, and subsequently cultured in fermentation medium under the same conditions for 20 h. As in our previous work [12], both the seed medium and fermentation medium consisted of 47 g/L glucose, 13 g/L yeast extract, 0.4 g/L MgSO_4_·7H_2_O, 3 g/L NH_4_Cl, 1 g/L KH_2_PO_4_ and 1 g/L K_2_HPO_4_ with initial pH 6.5. The culture conditions for *G. candidum* ZJPH1704 strain and *R. erythropolis* XS1012 see supporting information “S1”.

#### 3.2.2. General Procedure for Asymmetric Bio-Reduction of BTAP to (*S*)-3,5-BTPE

After 20 h culture of *C. tropicalis* 104, cells were harvested by centrifugation under 4500 rpm for 10 min at 4 °C, and washed twice with 10 mL potassium phosphate buffer (0.2 M, pH 8.0). Afterward, 300 g/L (wet cell weight) of the collected cells were resuspended in 10 mL potassium phosphate buffer (0.2 M, pH 8.0), then we then conducted the BTAP reductive reaction with 50 g/L maltose as cosubstrate, as in our previous work [12]. All trials were performed in triplicate.

#### 3.2.3. Investigation of Cofactor Specificity

Cofactor specificity of carbonyl reductase from *C. tropicalis* 104 was investigated as follows: assay was conducted at 30 °C by measuring the variation in the absorbance at 340 nm. The definition of enzyme activity was that 1 μmol of NADH or NADPH produced or consumed per minute under the assay conditions. The molar extinction coefficient of NAD(P)H was 6220 L·mol^−1^·cm^−1^. The protein concentration was determined by the Bradford method according to the report [36]. All trials were performed in triplicate.

#### 3.2.4. Assays of Intracellular NADH Concentration

First, the extraction of NADH proceeded. One milliliter of bio-converted solution was mixed with 1 mL pre-cooled (−20 °C) extractive solution comprised of 50% (*v*/*v*) absolute ethyl alcohol and 50% (*v*/*v*) 2.0 M KOH for quitting the reaction, and the mixture-containing tubes were immediately put into 70 °C water-bath for 7 min to obtain NADH. Then, after centrifugation at 12,000 rpm and 4 °C for 10 min, the supernatants were collected as samples for further assay. Based on literature [34], samples were detected at 570 nm and 30 °C, and checked every minute for 10 min. The slope of time-varying absorbance could be worked out. For example, in one of the tests, the slope of the control was 0.0109 and that of the test group was 0.0311 (see Figure S1b,c). Then the slope was put into a NADH standard curve, such as the curve in one of the tests that y = 0.0009x + 0.0049 (Figure S1a) to get the intracellular NADH concentration. All trials were performed in triplicate.

#### 3.2.5. Assays of Membrane Permeability

In 10 mL potassium phosphate buffer (0.2 M, pH 8.0) which contained 1% (*w*/*v*) NADES, 300 g/L (wet cell weight) of *C. tropicalis* 104 cells was resuspended in the buffer and hatched at 200 rpm and 30 °C. After a 24 h hatch, the cell-containing buffer was centrifuged at 8000 rpm, and the collected supernatant was detected at OD_260 nm_ and OD_280 nm_ to estimate the leaked nucleic acid and protein content, respectively [48]. All trials were performed in triplicate.

#### 3.2.6. Assays of Cell Viability

By detecting the amount of glucose consumed by cells pretreated in various NADES-containing buffer systems, the viability of *C. tropicalis* 104 cells was assessed. The operation was as follows: 300 g/L (wet cell weight) cells were pre-incubated at 30 °C, 200 rpm for 24 h in various NADES (1%, *w*/*v*)-phosphate buffer (0.2 M, pH 8.0) medium and in the presence of 200 mM BTAP or not, then treated cells were collected and hatched in 30 g/L glucose solution at 30 °C, 200 rpm for 3 h. Then, the residual glucose concentrations in the glucose solution were assayed by using a biological sensing analyzer. All trials were performed in triplicate.

#### 3.2.7. GC Analysis for (*S*)-3,5-BTPE and BTAP

After reaction, 40 mL ethyl acetate, which contained 4.4 mM dodecane, was added into the reaction system to terminate the reaction, and extract the resulting (*S*)-3,5-BTPE and residual BTAP. Then, 1 mL extraction-containing ethyl acetate was taken and dehydrated with anhydrous sodium sulfate. The produced (*S*)-3,5-BTPE and residual BTAP were detected on Agilent GC-7820A system with a flame ionization detector [36]. GC chiral column type was Varian CP-Chirasil-Dex (25 m × 0.25 mm × 0.25 μm, *df* = 0.25).

The detection conditions: the injection volume was 1 μL, and the split ratio was 15:1. Nitrogen carrier gas flowed at 2 mL·min^−1^ flux. The injection port and detector both were at 250 °C, and the column temperature was maintained at 80 °C for 2 min, then risen up to 125 °C at the rate of 5 °C·min^−1^. The retention times were 4.1 min for BTAP, 8.8 min for dodecane, 10.1 min for *(S)*-3,5-BTPE, and 10.6 min for (*R*)-3,5-BTPE. All trials were performed in triplicate.

## 4. Conclusions

In this study, the (*S*)-3,5-BTPE production mediated by crude *C. tropicalis* 104 in ChCl:T -containing micro-aerobic catalytic medium system, led to an exalting increase in the ratio of S/C without genetic manipulation for target carbonyl reductase activity of cells, addition of exogenous cofactor, or more amount demand of co-substrate for coenzyme regeneration. It was found that the enhancement of catalytic efficiency is relevant to the fortifying of intracellular cofactor recycle in *C. tropicalis* 104 resting cells by limiting oxygen supply during the bio-reductive reaction. Moreover, the capacity of introduced ChCl:T NADESs into reaction system was multifunctional for not only increasing the cell membrane permeability of *C. tropicalis* 104, but also declining the cytotoxicity of BTAP to biocatalyst, and thus improving bio-reductive efficiency. It is the first report on establishing and exploiting a feasible integrated strategy of altering respiratory pattern of resting whole-cell catalyst, and constructing a multifunctional NADES-containing reaction system for high-efficient synthesis of chiral aromatic alcohol. The developed combinational strategy also provides a novel “energy-saving” approach to improve the catalytic efficiency of (*S*)-3,5-BTPE production, and valuable insight for the development of asymmetric reduction by whole-cell biocatalyst.

## Figures and Tables

**Table 1:** Investigation of the cofactor specificity.

Substrate	NADH	NADPH	Specific Activity (U/g)
+	+	−	0.37 ± 0.04
+	−	+	none

Reaction conditions: 1 mL cell-free extract (24 mg of total protein) was added into 1 mL potassium phosphate buffer (0.2 M, pH 8.0) containing 50 mM BTAP, with the addition of 50 g/L maltose, 0.3 mM NADH or NADPH. + reagents or chemicals added, − not added.

**Table 2:** Assays of yield and NADH content under oxygen-deficient environment.

Group	NADH Content μmol/g Cell (DCW) ^b^	Yield (%) ^a^	ee (%) ^a^
Control	26.9 ± 0.01	41.54 ± 0.58	>99.9
Oxygen-deficient environment	40.3 ± 0.33	90.47 ± 0.54	>99.9

Reaction conditions: ^a^ 10 mL potassium phosphate buffer (0.2 M, pH 8.0), 300 g/L cells (wet cell weight), 50 mM BTAP, 50 g/L maltose, 30 °C, 200 rpm, in conical flask without or with stopper, reaction for 6 h. ^b^ After 6 h reaction and cells separation from reaction system, refer to the “3.2.4” method, the intracellular NADH was extracted and was put into 1800 μL bicine buffer (0.1 M, pH 8.0) containing 200 μL EDTA (Ethylene diamine tetraacetic acid, 40 mM), 100 μL MTT (3-(4,5-dimethyl-2-thiazolyl)-2,5-diphenyl-2-*H*-tetrazolium bromide, 12.068 mM), 75 μL absolute ethyl alcohol, 200 μL PES (Phenazine ethosulfate), and 100 U ADH (Alcohol dehydrogenase). The mixture was detected at 570 nm under 30 °C for 10 min.

**Table 3:** Yield of different species of strain under oxygen-deficient environment.

	Control		Oxygen-Deficient Environment	
Catalyst	Yield (%)	ee (%)	Yield (%)	ee (%)
*C. tropicalis* 104 ^a^	68.8 ± 3.54	>99.9	91.3 ± 1.84	>99.9
*G. candidum* ZJPH1704 ^b^	2.5 ± 1.23	66.9 ± 0.84	60.4 ± 2.91	>99.0
*R. erythropolis* XS1012 ^c^	78.7 ± 1.69	>99.9	74.4 ± 3.55	>99.9

Reaction conditions: ^a^ 10 mL potassium phosphate buffer (0.2 M, pH 8.0), 300 g/L cells (wet cell weight), 50 mM BTAP, 50 g/L maltose, reaction at 30 °C, 200 rpm for 24 h, under oxygen-deficient environment or not. ^b^ 10 mL Tris-HCl buffer (0.2 M, pH 8.0), 200 g/L cells (wet cell weight), 50 mM BTAP, 50 g/L glucose, reaction at 30 °C, 200 rpm for 24 h, under oxygen-deficient environment or not. ^c^ 10 mL Na_2_HPO_4_-KH_2_PO_4_ buffer (0.2 M, pH 8.0), 250 g/L cells (wet cell weight), 20 mM BTAP, 50 g/L glucose, reaction at 30 °C, 200 rpm for 24 h, under oxygen-deficient environment or not.

## Supplementary Materials

The following are available online, S1: Microorganism and Culture Conditions, Figure S1: (a) Standard curve of NADH; (b) OD_570 nm_ variation of sample in 6 h under oxygen-abundant condition; (c) OD _570 nm_ variation of sample in 6 h under oxygen-deficient condition. Figure S2: Chiral analysis of the bio-converted sample: (a) GC analysis for standards of BTAP, *(S)*-3,5-BTPE and *(R)*-3,5-BTPE with chiral Varian CP-Chirasil-Dex CB column; (b) GC chiral analysis of the bio-converted sample by *C. tropicalis* 104; (c) GC chiral analysis of the bio-converted sample by *G. candidum* ZJPH1704; (d) GC chiral analysis of the bio-converted sample by *R. erythropolis* XS1012. Figure S3: ^1^H NMR Spectroscopy of the product by *C. tropicalis* 104. Figure S4: ^13^C NMR Spectroscopy of the product by *C. tropicalis* 104. Figure S5: ^1^H NMR Spectroscopy of ChCl: T (1:1).
