# Supplementary material for: High-Efficient Production of (S)-1-[3,5-Bis(trifluoromethyl)phenyl]ethanol via Whole-Cell Catalyst in Deep-Eutectic Solvent-Containing Micro-Aerobic Medium System
Source: Molecules. 2020 Apr 17;25(8):1855. doi: 10.3390/molecules25081855 (PMC7221904; doi:10.3390/molecules25081855)
Supplement: Supplementary file 1 [file molecules-25-01855-s001.pdf]

Supplementary Materials

High-efficient production of (*S*)-1-[3,5-bis(trifluoromethyl)phenyl]ethanol via whole-cell catalyst in deep-eutectic solvent-contained micro-aerobic medium system

Zhiren Zhu, Shunde Bi, Ning Ye, Pu Wang *

College of Pharmaceutical Science, Zhejiang University of Technology, Hangzhou 310014, China; zhuzhiren619@163.com; bishundezjut@163.com; itsukiyening@gmail.com

* Correspondence: wangpu@zjut.edu.cn (P. W.); Tel.: +86-571 -88320389

S1. Microorganism and Culture Conditions

For *Geotrichum candidum* ZJPH1704 (GenBank No. MG214158), the seed medium consisted of: glucose 15 g/L, peptone 20 g/L, yeast extract 10 g/L, $(NH_4)_2SO_4$ 2 g/L, NaCl 1 g/L, $MgSO_4{\cdot}7H_2O$ 0.5 g/L, KH_2PO_4 2 g/L, with initial pH of 6.5. The strain was cultured in seed medium for 12 h under 200 rpm and 30 °C, and then in fermentation medium for 24 h under the same conditions. The fermentation medium consisted of: glucose 24.45 g/L, peptone 15.75 g/L, $(NH_4)_2SO_4$ 21.39 g/L and $CaCl_2$ 0.11 g/L with pH 6.5.

For *Rhodococcus erythropolis* XS1012 (GenBank No. KJ000875), the seed medium consists of: glucose 15 g/L, peptone 7.5 g/L, yeast extract 6 g/L, $(NH_4)_2SO_4$ 3 g/L, NaCl 0.75 g/L, $MgSO_4{\cdot}7H_2O$ 0.75 g/L and KH_2PO_4 1.5 g/L with pH 6.5. The strain was cultured in seed medium for 24 h under 200 rpm and 30 °C, and then in fermentation medium for 48 h under the same conditions. The ingredients of fermentation medium are the same as the seed medium.

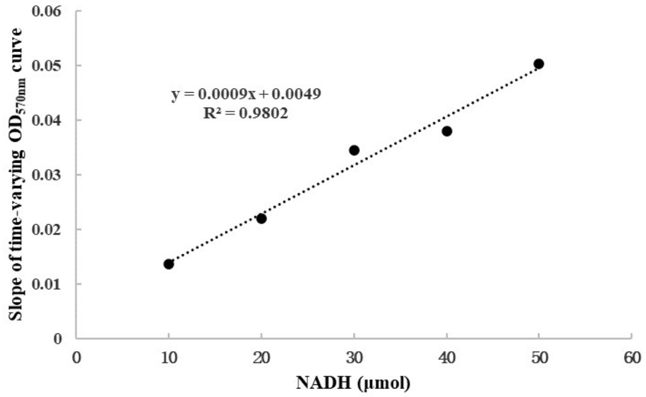

(a)

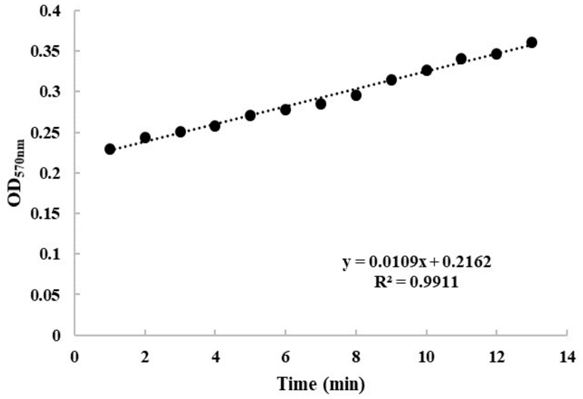

(b)

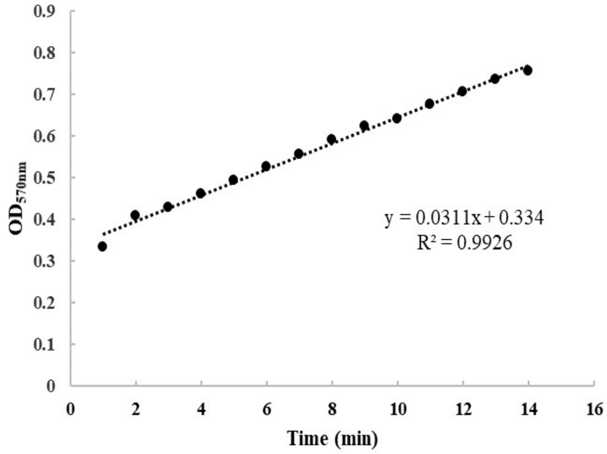

(c)

Figure S1: (a) Standard curve of NADH. (b) OD_{570nm} variation of sample in 6 h under oxygen-abundant condition. (c) OD_{570nm} variation of sample in 6 h under oxygen-deficient condition.

S2. Assays of the Intracellular NADH Concentration

Put the slopes of (b) and (c) into the standard curve (a) to figure out NADH content of corresponding samples. The ratio of NADH content/cell dry weight is the NADH concentration.

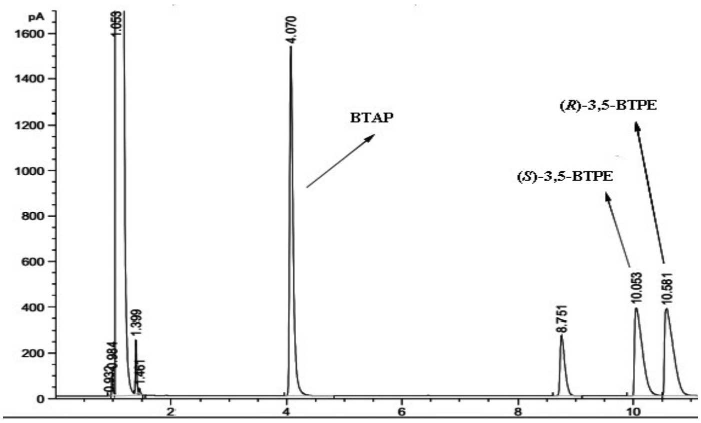

(a)

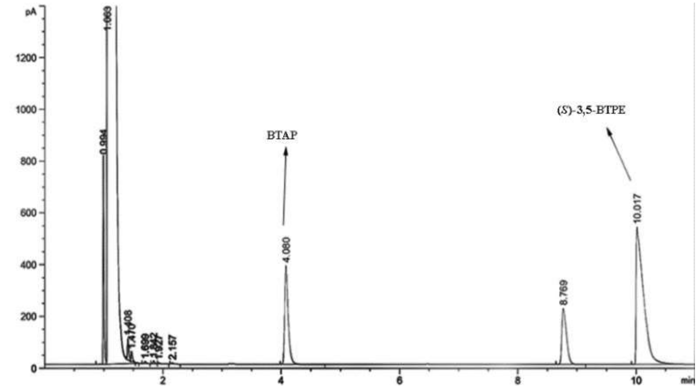

(b)

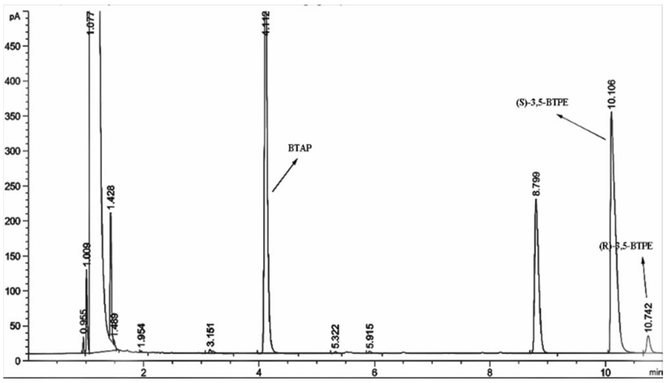

(c)

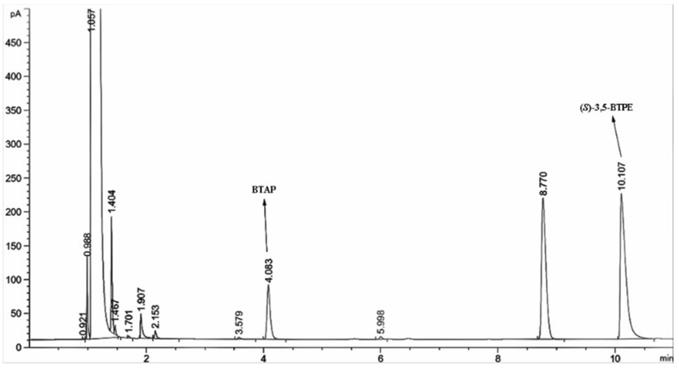

(d)

Figure S2: Chiral analysis of the bioconverted sample. (a) GC analysis for standards of BTAP, (S)-3,5-BTPE and (R)-3,5-BTPE with chiral Varian CP-Chirasil-Dex CB column. (b) GC chiral analysis of the bioconverted sample by *C. tropicalis* 104. (c) GC chiral analysis of the bioconverted sample by *G. candidum* ZJPH1704. (d) GC chiral analysis of the bioconverted sample by *R. erythropolis* XS1012.

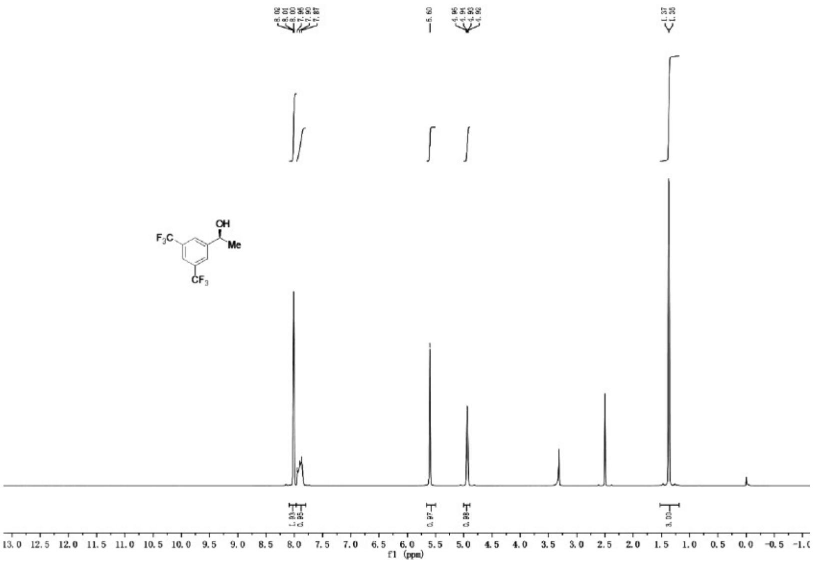

Figure S3: ^{1}H NMR Spectroscopy of the product by *C. tropicalis* 104.

^{1}H NMR (600 MHz, DMSO-d_6) δ 8.01 (t, J = 5.0 Hz, 2H), 7.97-7.81 (m, 1H), 5.60 (s, 1H), 4.94 (q, J = 6.8 Hz, 1H), 1.37 (d, J = 6.0 Hz, 2H).

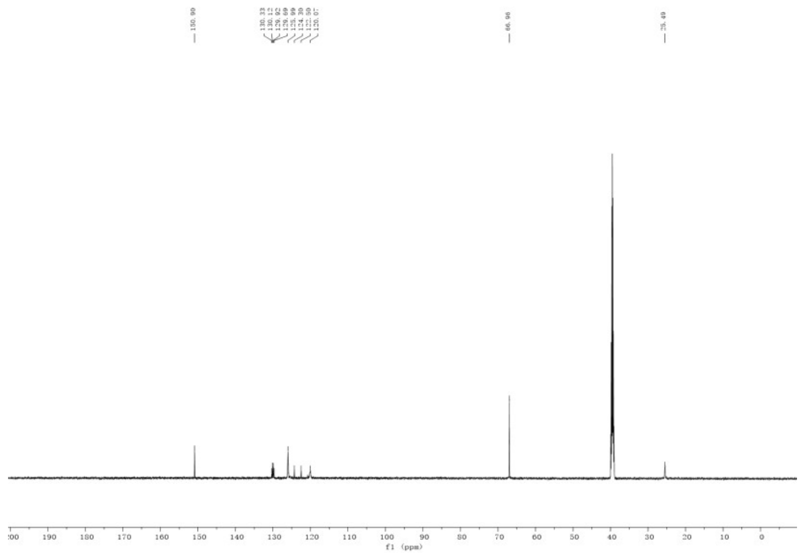

Figure S4: ^{13}C NMR Spectroscopy of the product by *C. tropicalis* 104.

^{13}C NMR (151 MHz, DMSO-d_6) δ 150.90, 130.01 (q, *J* = 34.2, 32.4 Hz), 125.99, 124.30, 122.50, 120.07, 66.96, 25.49.

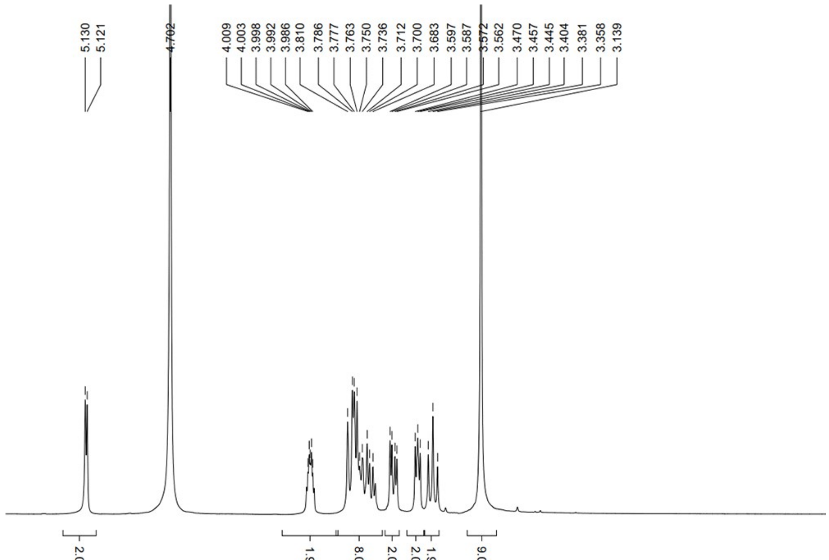

Figure S5: ^{1}H NMR Spectroscopy of ChCl: T (1:1)

^{1}H NMR(400 MHz, D_2O): δ=5.126 (d, 2H, J=3.6 Hz), 3.998 (m, 2H, J= 2.3 Hz), 3.743 (m, 8H), 3.579 (dd, 2H, J=4.7 Hz), 3.457 (t, 2H, J=5 Hz), 3.381 (t, 2H, J=9.2 Hz), 3.139 (s, 9H)
